# Pancreatic Cancer: 80 Years of Surgery—Percentage and Repetitions

**DOI:** 10.1155/2016/6839687

**Published:** 2016-10-25

**Authors:** Birgir Gudjonsson

**Affiliations:** The Medical Clinic, Álfheimum 74, 104 Reykjavik, Iceland

## Abstract

*Objective*. The incidence of pancreatic cancer is estimated to be 48,960 in 2015 in the US and projected to become the second and third leading causes of cancer-related deaths by 2030. The mean costs in 2015 may be assumed to be $79,800 per patient and for each resection $164,100. Attempt is made to evaluate the results over the last 80 years, the number of survivors, and the overall survival percentage.* Methods*. Altogether 1230 papers have been found which deal with resections and reveal survival information. Only 621 of these report 5-year survivors. Reservation about surgery was first expressed in 1964 and five-year survival of nonresected survivors is well documented.* Results*. The survival percentage depends not only on the number of survivors but also on the subset from which it is calculated. Since the 1980s the papers have mainly reported the number of resections and survival as actuarial percentages, with or without the actual number of survivors being reported. The actuarial percentage is on average 2.75 higher. Detailed information on the original group (TN), number of resections, and actual number of survivors is reported in only 10.6% of the papers. Repetition occurs when the patients from a certain year are reported several times from the same institution or include survivors from many institutions or countries. Each 5-year survivor may be reported several times.* Conclusion*. Assuming a 10% resection rate and correcting for repetitions and the life table percentage the overall actual survival rate is hardly more than 0.3%.

## 1. Introduction

Resections for pancreatic adenocarcinoma which Whipple et al. [1] initiated after earlier attempts by Codivilla [2] and Kausch [3] have now been carried out for 80 years. Opinions still differ as to the results. Some authors claim a survival percentage of up to 22% and are widely quoted and extol the benefits and success of resections [4] while others doubt that anyone survives pancreatic cancer [5].

## 2. Incidence, Economics

The incidence of pancreatic cancer has been estimated at 48,960 in 2015 in the US and is the fourth leading cause of death from cancer for both sexes [6]. It is projected to increase to 62,000 in the year 2020 and to 88,000 for both sexes in 2030 and to surpass breast, prostate, and colorectal cancers to become the second and third leading causes of cancer-related deaths by 2030 [7].

The cost of treatment of pancreatic cancer is of concern in many countries. O'Neill and colleagues studied the total direct medical cost of patients 66 years and older who were diagnosed from 2000 to 2007 in the US. The mean total direct cost was $65,500, for resectable locoregional disease cost was $134,700, and for unresectable locoregional or distant disease cost was $65,300 and $49,000, respectively [8]. Lea and Stahlgren had earlier pointed out the difference in the cost of resections versus bypass [9].

Assuming 2.5% inflation over 8 years, the mean cost in 2015 would be $79,800, for resections $164,100, for unresectable (or bypasses) disease $79,500, and for distant disease $59,700. With the estimated number of patients in 2015 the overall cost would be close to $4 billion.

## 3. Methods and Evaluation of Results

There is a growing concern that reports of success in medical research are inflated [10].

Here an attempt is made to evaluate the results over the last 80 years, the number of survivors, and the overall survival percentage.

This author has continued to scrutinize the literature on surgery from the onset, initially using the* Index Medicus* and then Ovid/PubMed until the end of 2014, with cross-references. Approximately 1230 papers have been found in 15 languages in approximately 200 journals from 44 countries which deal with resections and reveal at least some survival information. A total of 40.5% of the papers originated in the US, 19.3% from Japan, 7.3% from Germany, 5.3% from Italy, and 4% from France and the UK. These have been inserted into a database.

Papers on the surgical aspects of pancreatic cancer differ as to the approach and the composition of the patient group and the method of reporting. A few emphasize only the technical aspects and the mortality with limited or no survival information and indiscriminately cover patients with various malignant and benign pathologies which may require pancreatoduodenal resection, but without clearly separating each pathology group or presenting separate survival information. Only papers with separate pathologic information on patients were selected for analysis for this paper. Analysis of the database reveals that, of these 1230 papers, 609 do not report any 5-year survivors, some seem to be mainly technical, and some report only up to a 3-year survival rate. A total of 621 papers report 5-year survivors and will be examined further in detail in this paper. Special attention has been paid to the origin of each paper, the time period each study covered, patient composition, the subset of patients used for calculations, and the statistical method used.

## 4. Reservation, Nonresected Survivors

The first reservation about the effect of surgery on this disease was expressed by Glenn and Thorbjarnarson [11] in 1964, again by Gallitano et al. [12] in 1968 whose only 5-year survivor was “nonresected,” and then strongly by Crile Jr. in 1970 [13], whose only survivor was also nonresected. He challenged the value of resection for pancreatic cancer, followed by Shapiro in 1975 [14]. Crile Jr.'s criticism was directed at the then high mortality rate and the survival calculations which were carried out and might count only those who survived the operation and in ignorance of the nonresected survivors.

The presence of nonresected survivors has been disputed [15], but it is a major issue in the debate on survival. It was first pointed out by Cattell and Young in 1957 [16] and, as above, later by Gallitano and Crile Jr. The data were summarized by the present author in a paper in 1995 [17] and a letter in 1996 [18]. In a review by the present author published in 1978 only 65 five-year survivors could be found in the literature, of whom 8 were nonresected [19]. In a review published in 1987, 165 survivors could be found, but 12 of these were nonresected [20]. In this review 41 reports have been found from 31 institutions in 12 countries, many from eminent institutions and renowned authors, thereof 17 from the US, two from Yale [19, 20], two from the University Texas MD Anderson [12, 21], and two from Harvard MGH [22, 23], as well as from the University of Chicago [24], the Dana Farber Cancer Clinic [25], and Thomas Jefferson [26], to mention a few.

Nonresected survival is a fact and should be kept in mind in assessing overall therapeutic results. Initially reports detailed the course of all patients diagnosed at a particular institution but in recent decades reports have concentrated only on resected patients, completely ignoring any nonresected survivors. Nonresected survivors would therefore not be found.

## 5. Survival Calculations

The survival percentage depends not only on the number of survivors but on the subset from which the number is calculated.

A few earlier studies started by examining the respective tumor registries and disclosed that only about 35–68% of patients in tumor registries had histologic confirmation. Survival calculations have been based on the original number of patients with histologic diagnoses at a particular institution, previously called the total number (TN), the approximately 80% of cases that were surgically explored, the cases that were resected, or location, size, or R status of the tumor, or even only those patients who survived the operation.

Overall survival success must be based on the original group diagnosed with pancreatic cancer (the TN or total number) and the number of survivors and not only on a small subgroup of the cases. Different methods of calculation have been used to enumerate the results, that is, actual versus the actuarial, projected, or estimated percentage.

Initially most papers revealed the TN, the number of resections, and simply the number of survivors, whereas later authors also presented actual percentage figures. In the late 1980s the papers started reporting only the number of resections and survival as actuarial percentages, usually calculated with the Kaplan-Meier method with or without the actual number of survivors being reported [27].

Sir Hill pointed out in his book in 1937 that when a “large number of patients is lost sight of” the outcome might be erroneously high. This warning is reemphasized in later editions [28]. In a frequently quoted paper 11 survivors out of 201 are claimed as proof of 22% survival [4].

As indicated in Table 1 the original number TN of patients studied in a report is only revealed in 90 or 14.5% of the papers and in these the actual number of survivors is stated in only 49. In the remaining 41 with a documented TN and actuarial calculation the actual number of survivors is stated in 17, in addition to life table curves. Detailed information on the original TN group studied, number of resections, and actual number of survivors is therefore reported in only 66 or 10.6% of all papers on pancreatic cancer. In the remaining 89.4% some form of estimate or calculation is required to assess survival percentage. In 531 papers there is no information on the original number of patients from which the number of resections was drawn, although in 102 of these the number of survivors is stated or confirmed by inquiry.

In 424 of these 531 reports with survival calculations by actuarial methods 378 are by the Kaplan-Meier method and 48 by other or unclear methods, though KM is also very likely. The number of survivors is stated in 147 of the reports or 34.6%, but not in the remaining 277 or 65.3%.

A total of 240 inquiries were sent to authors where the actual number of patients was not reported and only 58 replies were obtained. The actual number of survivors with actuarial calculation is therefore known in 205 of the 424 reports or 48.3%. The actuarial and actual percentage figures can therefore be compared, as demonstrated previously [29, 30], and reveal that the actuarial percentage is on average 2.75 higher than the actual percentage. This figure has therefore been used to estimate the number of survivors and the survival percentage in the relevant studies where only the actuarial percentage has been published.

The resection rate has been debatable and varies and can only be assessed accurately if the original group is large and well defined. Tertiary referral centers cannot know the size of the original group from which their resection group is drawn. Of the studies published in the last 5 years, 156 of 161 or 97% report only the number of resections and the percentages. In an earlier study by this author the resection rate was 10.8%. In earlier US studies [31, 32] the rate was, respectively, 8.4% and 12%. In 2 European nationals [33, 34] the rate was from 8% to 12% over the last 5 years. In a recent report from the surgical service at a European university hospital [35] the resection rate was 11.6%. It is therefore practical to assume that the resection rate is 10% in the studies where the original TN of the group is not reported in order to estimate the TN accordingly and divide the percentage by 2.5–3 where the actuarial KM only has been published.

After totaling the numbers in the 621 studies with survivors with the above correction, but without further adjustment, the TN comes to 1,731,834, the number of resected patients comes to 162,207, and the number of survivors comes to 11,300, for an apparent survival percentage of 0.77%.

After totaling the number of patients in all the 1230 reports, the original TN comes to 3,188,543, the number of resected patients to 284,298, and the number of survivors to 11,330. The overall survival percentage would then be only 0.45%.

## 6. Repetitions

Repetition of reporting the same survivors in different papers was first pointed out in 1978 [17]. It occurs in various ways, such as when papers include survivors from many different institutions or known databases in a specific country or even when a study includes patients from many countries. Thus 92 of the 620 studies with 5-year survivors are from many institutions in a specific country or 14.8% and 10 of these from many countries or 1.6%.

**Figure 1:**
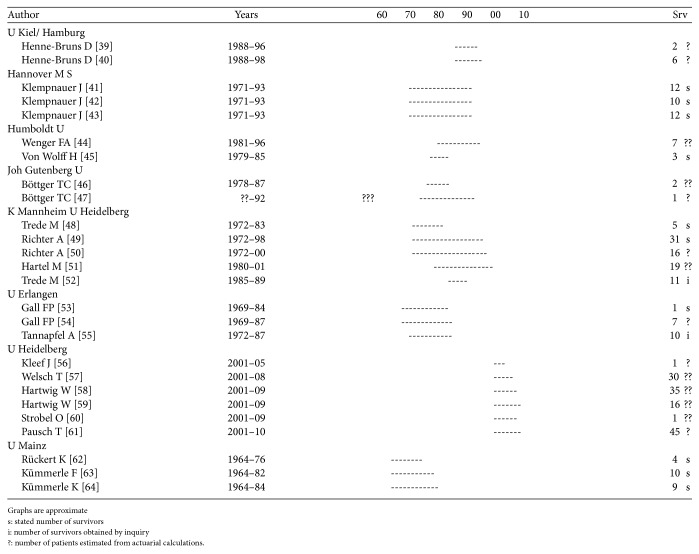
Repetitions Germany sample. See [40–65].

**Figure 2 fig2:**
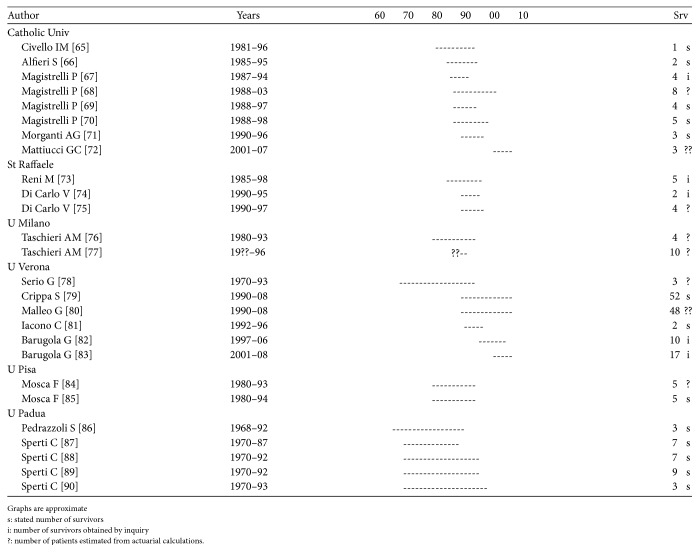
Repetitions Italy sample. See [66–91].

**Figure 3 fig3:**
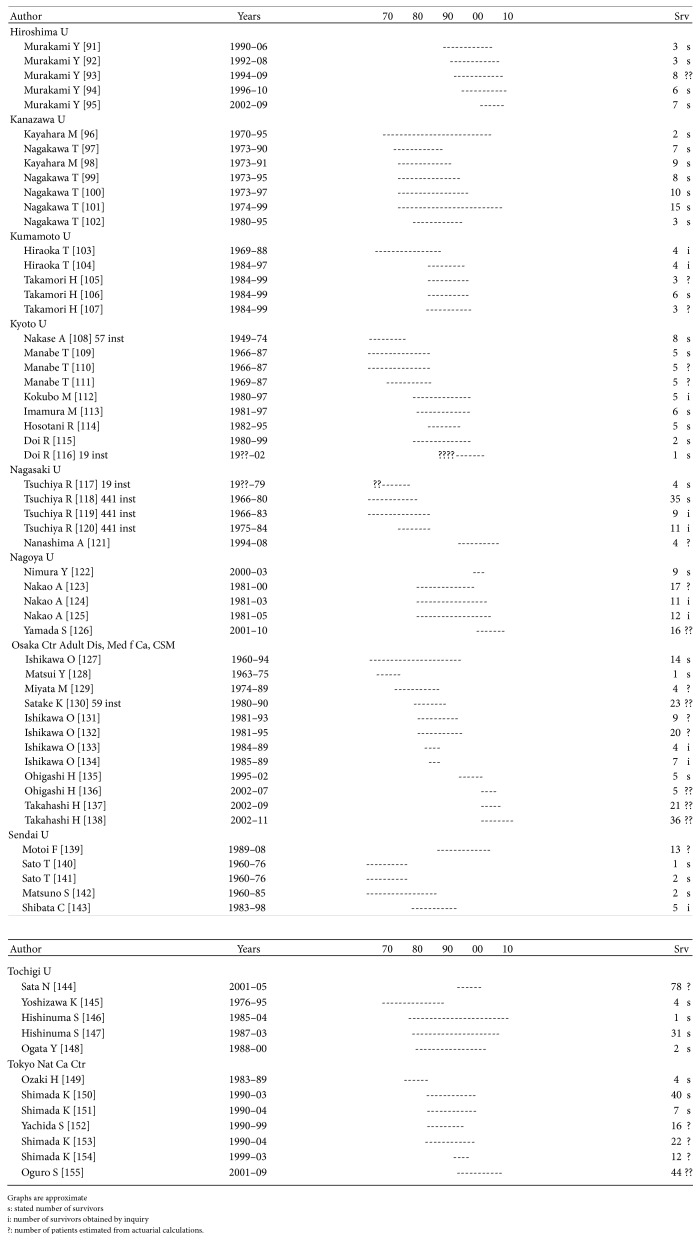
Repetitions Japan sample. See [92–156].

**Figure 4 fig4:**
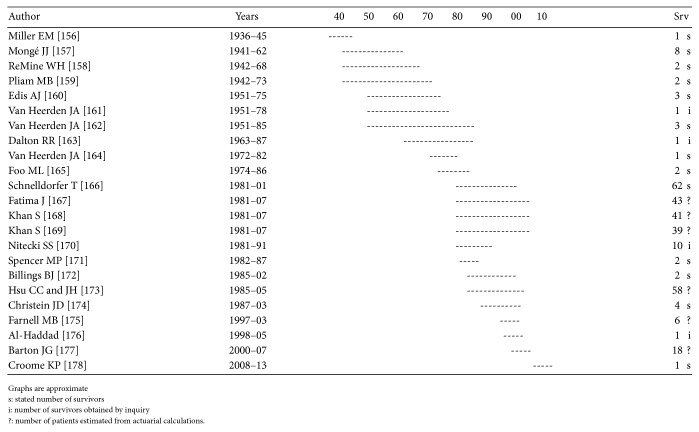
Repetitions Mayo Clinic. See [157–179].

**Figure 5:**
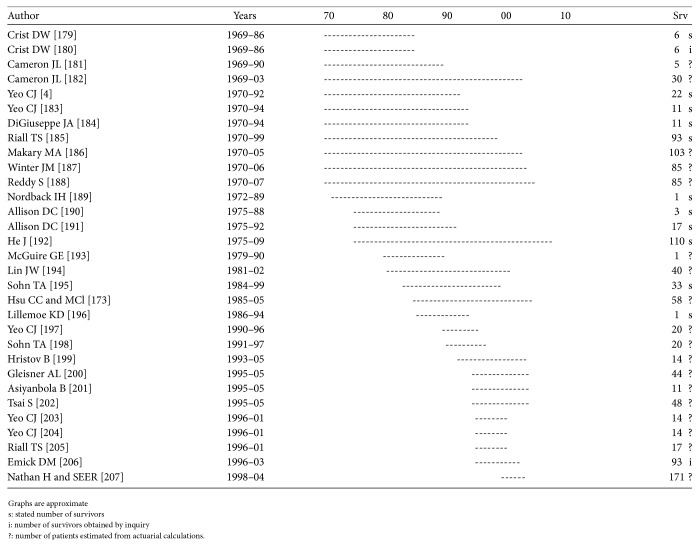
Repetitions Johns Hopkins University. See [38, 180–207].

Repetition occurs, though mainly when the patient population and survivors from a certain year are reported several times from the same institution. As can be seen in Figures 1 [Fig fig2] [Fig fig3] [Fig fig4]–5, repetition has occurred up to 6–8 times in Germany, Italy, and Japan and up to 20 times in the US.

Examination of reports from a single institution covering the entire study period and stating the number of survivors and then adding up the number of patients from all the studies, including those with an estimated number of survivors, reveals that the total number reported is over 10 times larger than the number reported in the studies with a documented number of survivors.

Each paper may at times reveal some new information but only infrequently is it disclosed that the patients have been reported before.

There is no scientific method to assess the number of repetitions accurately but each reported 5-year survivor and thereby respective resection and the TN seems to be reported 3–5 times. Dividing the number of reported survivors and respective resections and TN by 4, the overall number of 5-year survivors is hardly more than 2,800, the number of resections 40,500, and the original TN number of patients 433,000.

Repetitions occur also in the “no-survivor” group of reports, but not as frequently. It may be assumed that all published reports with or without survivors are drawn from a TN of approx. 1,000,000 patients and with fewer than 3,000 survivors, of whom a significant number were nonresected, meaning that the overall survival rate was no more than approximately 0.3%.

## 7. Mortality, Positive Margins, and Nodes

Mortality during the first 20 years, 1945–1965, was on average 25.2% with a single report of 62.5%. During the next 20 years or up to 1984 mortality was on average 19.9% with the highest rate at 52%. In 1985–1994 it lowered to 9.8%. In subsequent 5-year periods mortality was reduced to 6.8% and then 4.6% and during the last 5 years 4% with a high of 33%. Aside from the 33%, the average is now 3.7%. The overall mortality rate has therefore greatly reduced.

The majority of surgeons in recent decades have reported the number of positive margins and nodes and numbers over 60–70% frequently quoted [36–38]. It is of great interest that even in the most experienced hands only 16% of cases were both margins and nodes negative [38].

Tumor cells can be found in the bone marrow in up to 50% of cases [39].

## 8. Discussion and Conclusion

Pancreatic cancer is thus both a costly and devastating disease and has usually spread beyond its boundaries at time of diagnosis and treatment and is thus a systemic disease. The literature on pancreatic surgery, while purporting to report the facts, is nevertheless inaccurate.

The use of actuarial calculation methods exaggerates the percentage and thereby the number of presumed survivors in a particular study.

Reporting the same patients repeatedly without any qualification gives a false impression of success.

Life table curves should be accompanied by the actual number of survivors. The course of nonresected patients should be studied.

Surgical skills are imperative for the care and palliation of pancreatic cancer patients including possible resections, but they have had only a minimal impact on the survival rate.

It is of importance for the medical profession that published results are indisputable.

## Figures and Tables

**Table 1:** Survival information.

Categories of reports	Number of reports	Reports with/without survivors	Reports with/without stated TN	Reports with actual survival calculations	Reports with actuarial calculations and stated number of survivors	Reports with actuarial calculation and survivors confirmed by inquiry	Reports with actuarial calculations and estimated number of survivors
TN number of reports	1230						
Reports with survivors		621					
Reports with stated TN of patients			90				
Actual survival calculations				49			
Actuarial calculations with stated number of survivors					17		
Actuarial calculation with survivors confirmed by inquiry						7	
Reports with estimated number of survivors							17
Reports with estimated TN of patients			531				
Actual survival calculations				102			
Reports with actuarial calculation and stated number of survivors					146		
Survivors confirmed by inquiry						57	
Estimated number of survivors							226
Reports without survivors		609					
